# Duloxetine for the Treatment of Chronic Low Back Pain: A Systematic Review of Randomized Placebo-Controlled Trials

**DOI:** 10.7759/cureus.15169

**Published:** 2021-05-22

**Authors:** Takashi Hirase, Jessica Hirase, Jeremiah Ling, Peggy H Kuo, Gilbert A Hernandez, Kayode Giwa, Rex Marco

**Affiliations:** 1 Orthopedics and Sports Medicine, Houston Methodist Hospital, Houston, USA; 2 Psychiatry, Houston Methodist Hospital, Houston, USA

**Keywords:** duloxetine, chronic pain, low back pain, pain management, lumbar spondylosis

## Abstract

This systematic review determines the efficacy and safety of duloxetine for chronic low back pain (CLBP). We queried the PubMed, SCOPUS, and Ovid MEDLINE databases. All level I and II randomized controlled studies published in the English language investigating the efficacy of duloxetine for chronic low back pain were included. Five studies (832 duloxetine-treated patients, 667 placebo-treated patients, and 41 duloxetine and placebo crossover analysis patients) were analyzed. One study was level I evidence and four studies were level II evidence. All five studies reported statistically significant improvements in more than one back-pain-specific clinical outcome score with duloxetine versus placebo. Four studies found that duloxetine 60 mg daily leads to one or more statistically significant improvements versus placebo in Brief Pain Inventory Severity (BPI-S) scores. All five studies found no significant difference in serious adverse events (AEs) between the duloxetine and placebo groups. One study found a higher rate of total AEs among the duloxetine 120 mg group versus the placebo group; however, the same study did not find a significant difference in total AEs among duloxetine 20 mg and 60 mg groups versus placebo. Duloxetine is a safe and effective first-line option for the treatment of CLBP. Current studies demonstrate that 60 mg taken once daily has the highest efficacy for reducing pain and disability while minimizing minor adverse effects. Further randomized controlled trials with long-term follow-up are necessary to determine its long-term effects.

## Introduction and background

Low back pain is a leading cause of disability in the United States with a lifetime prevalence of up to 80% [1-3]. There is a tremendous economic burden associated with this condition with an annual treatment cost of up to $200 billion and an estimated 149 million workdays missed per year within the United States [4-5]. Chronic low back pain (CLBP) is defined as pain that persists for greater than three months and has been associated with a significant increase in the use of spine surgery, spinal injections, and opioids in the last two decades [4-7].

The etiology of CLBP appears to be multifactorial and is not clearly understood. Degenerative lumbar spondylotic changes have been proposed to be a common etiology associated with this condition, which leads to neuronal hyperexcitability, hypersensitization, and increased inflammatory factors within the central nervous system [8-9]. Recent clinical guidelines recommend non-operative treatment consisting of exercise and non-steroidal anti-inflammatory drugs (NSAIDs) as first-line treatment for non-specific CLBP [10]. However, exercise is often ineffective, and gastrointestinal, renal, and cardiac adverse events have been associated with NSAIDs, particularly with chronic use [10-12]. The moderate use of opioids and muscle relaxants has also been recommended for short-term treatment; however, this has significant limitations due to neurologic, psychosocial, and gastrointestinal side effects [13-14]. Due to these limitations, clinicians often resort to invasive treatment methods, including spinal injections and surgical interventions prior to the completion of a lengthy trial of non-operative management [10,15].

Duloxetine is a serotonin-norepinephrine reuptake inhibitor (SNRI) that is often used for major depressive disorder (MDD) and generalized anxiety disorder (GAD) [16-17]. This medication inhibits the neuronal reuptake of serotonin and norepinephrine and has recently been shown to additionally be effective for the treatment of chronic neuropathic pain and fibromyalgia [18-19]. Since then, multiple randomized controlled trials have been performed evaluating the use of duloxetine in chronic low back pain with favorable results [20-24]. The purpose of this analysis was to develop a comprehensive, systematic review of randomized placebo-controlled trials in the current literature that investigates duloxetine for the treatment of CLBP. The authors hypothesized that duloxetine is a safe and effective pharmacological intervention for the treatment of CLBP.

## Review

Methods

Registration was completed for this systematic review with the International Prospective Register of Systematic Reviews (PROSPERO) on December 11, 2020. No similar prior systematic reviews or meta-analyses were identified within PROSPERO. The protocol described in the Preferred Reporting Items for Systematic Reviews and Meta-Analyses (PRISMA) guidelines were utilized to conduct the search [25].

Two authors conducted separate searches using the following medical databases on December 09, 2020: PubMed (1966-present), SCOPUS (1966-present), and Ovid MEDLINE (1946-present). To ensure a stringent search strategy of relevant literature, keywords including “duloxetine,” “back,” and “pain” were combined with Boolean operators to develop a search protocol. To further minimize unintentional exclusion of relevant studies, two authors performed a separate hand search of the included references.

All level I and II evidence randomized placebo-controlled trials (as defined by the Oxford Centre for Evidence-Based Medicine [CEBM]) published in the English language that investigated the efficacy and safety of duloxetine for the treatment of chronic low back pain were included [26].

Studies were excluded if they were studies of non-chronic low back pain, studies with concomitant MDD, non-placebo controlled studies, and non-randomized controlled studies, including cadaveric studies, basic science studies, animal studies, diagnostic studies, economic studies, prognostic studies, letters to editors, review articles, editorials, and surveys. Only one study was retained in the situation of duplicate studies from the same author(s) and/or institution(s) reporting on overlapping subjects: the longest follow-up, the highest level of evidence, the most pertinent outcome scores investigated, and the largest number of subjects.

Three authors independently reviewed all studies using a previously recommended methodology [27]. The level of evidence (CEBM), study design, and methodological quality of each study was graded using the Modified Coleman Methodology Scores (MCMS) [26,28]. For each included study, the Grading of Recommendations Assessment, Development and Evaluation (GRADE) score and the overall Strength-of-Recommendation Taxonomy (SORT) scores were calculated [29-30]. Patient demographics, including age, gender, diagnosis, adverse events, patient-reported outcome scores, and the study authors’ overall conclusion, were extracted from each study. WebPlotDigitizer version 4.4 (Ankit Rohatgi, Pacifica, CA, USA, https://automeris.io/WebPlotDigitizer) was utilized to best estimate the reported data using prior described methods for data extracted from digital plots [31-32]. If the included studies were too heterogeneous, with heterogeneity in study participants, interventions, and/or outcomes, a meta-analysis would not be performed and a systematic review with best-evidence synthesis would be chosen as the synthetic review type. 

The Statistical Package of the Social Sciences (SPSS) statistical software (Version 25.0; IBM Corp., Armonk, NY) was utilized for statistical analysis. The chi-square test was used to analyze categorical data and the two-tailed student t-test was used to analyze continuous data. A p-value < 0.05 was considered statistically significant.

Three authors used the Revised Cochrane Risk-of-Bias tool for randomized trials (RoB 2) tool to perform a risk-of-bias assessment of each included study [33].

Results

**Figure 1:**
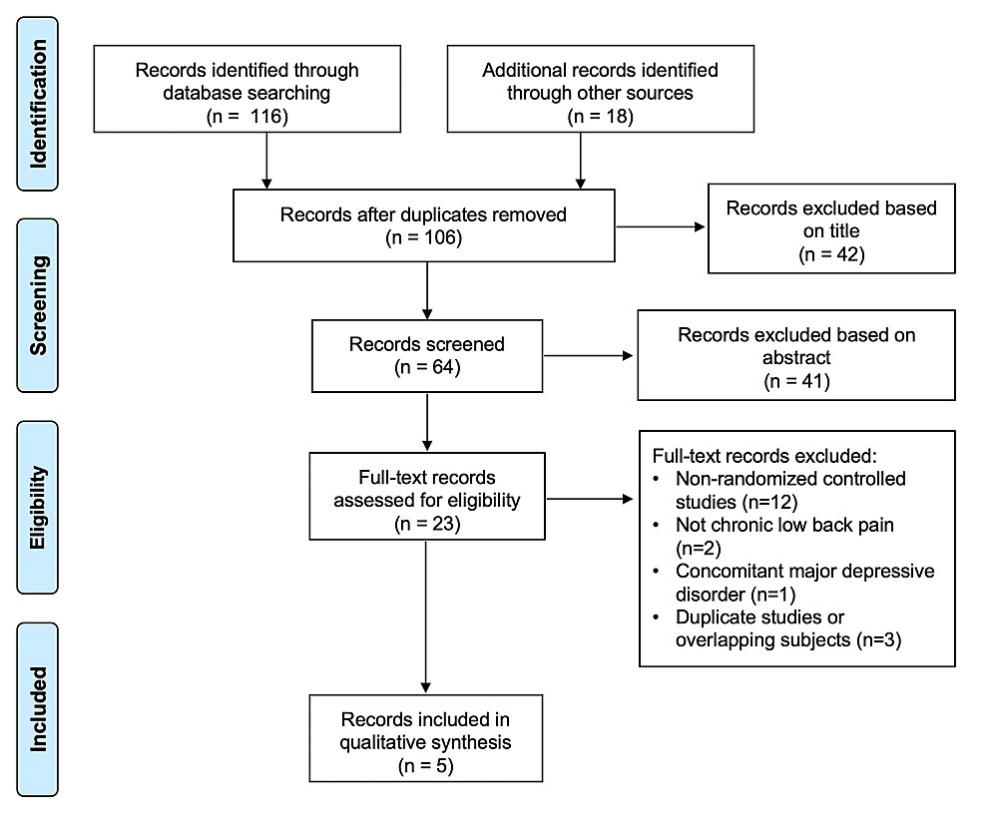
The Preferred Reporting Items for Systematic Review and Meta-Analysis (PRISMA) flowchart showing the application of selection criteria to the studies identified with the search strategy

One-hundred-and-thirty-four studies were identified during the preliminary search with 28 found to be duplicates. Of the remaining 106 studies, five met all inclusion and exclusion criteria (Figure 1).

One study was level I evidence and four studies were level II evidence. According to MCMS, four studies were rated as excellent (scores between 85 and 100) and one study was rated as good (scores between 70 and 84) [26]. The overall SORT and GRADE scores were A [29,30]. According to RoB2, the overall risk of bias was low for all five studies (Table 1) [33].

**Table 1:** Study demographics included in the analysis Rob2 = revised Cochrane Risk-of Bias tool for randomized trials; MCMS = Modified Coleman Methodology Score; SD = standard deviation; CLBP = chronic low back pain; NR = not recorded; NSAID = non-steroidal anti-inflammatory; QTF = Quebec Task Force *p<0.05 across treatment groups

Study	Skljarevski et al. 2009				Skljarevski et al. 2010a		Skljarevski et al. 2010b		Konno et al. 2016		Shuckro et al. 2017
Type of Study	Prospective, randomized, double-blind, placebo-controlled trial				Prospective, randomized, double-blind, placebo-controlled trial		Prospective, randomized, double-blind, placebo-controlled trial		Prospective, randomized, double-blind, placebo-controlled trial		Prospective, randomized, double-blind, placebo-controlled crossover trial
Level of Evidence	II				II		II		I		II
Clinical Trial Registry No.	NCT00408876				NCT00424593		NCT00767806		NCT01855919		NC01166048
Countries	Brazil, France, Germany, Mexico, Netherlands				Brazil, France, Germany, Mexico, Netherlands		United States, Brazil, Germany, Netherlands, Poland, Russia, Spain		Japan		Austria
Rob2 Risk of Bias	Low				Low		Low		Low		Low
MCMS	92				90		88		94		82
Dates of Cohort	2006-2007				2007-2008		2009		2013-2014		2010-2013
Groups	Duloxetine 20 mg	Duloxetine 60 mg	Duloxetine 120 mg	Placebo	Duloxetine 60-120 mg	Placebo	Duloxetine 60 mg	Placebo	Duloxetine 60 mg	Placebo	Crossover (Duloxetine 120 mg/Placebo)
No. of subjects	59	116	112	117	115	121	198	203	232	226	41
Age, mean (SD)	52.9 (12.8)	53.3 (14.7)	54.9 (14.8)	54.0 (13.5)	51.8 (14.9)	51.2 (13.5)	54.9 (13.7)	53.4 (14.2)	60.0 (13.2)	57.8 (13.7)	57.9 (13.4)
Male, n (%)	23 (39.0)	49 (42.2)	47 (42.0)	53 (45.3)	44 (38.3)	48 (39.7)	80 (40.4)	75 (36.9)	115 (50.0)	104 (46.0)	20 (48.8)
Weight (kg), mean (SD)	84.9 (14.2)	81.2 (15.3)	82.6 (18.6)	81.9 (17.4)	76.2 (14.7)	75.9 (13.9)	78.3 (15.8)	79.4 (14.7)	63.6 (12.8)	63.2 (13.4)	80.5 (18.3)
Duration of CLBP (years), mean (SD)	12.5 (11.7)*	10.5 (11.1)*	13.9 (13.0)*	10.3 (9.5)*	8.8 (8.8)	9.5 (8.6)	8.3 (8.2)	8.7 (9.0)	9.8 (10.1)	10.3 (10.6)	1.5 (NR)
NSAID use, n (%)	22 (37.3)	51 (44.0)	51 (45.5)	43 (36.8)	35 (30.4)	39 (32.2)	NR	NR	0 (0.0)	0 (0.0)	12 (29.2)
History of CLBP surgery, n (%)	0 (0.0)	0 (0.0)	0 (0.0)	0 (0.0)	7 (6.1)	12 (9.9)	NR	NR	0 (0.0)	0 (0.0)	NR
QTF class 1, n (%)	43 (75.4)	88 (80.0)	80 (75.5)	88 (80.7)	76 (67.9)	74 (64.4)	173 (89.2)	168 (84.8)	NR	NR	NR

These five studies contained: 1540 patients treated with (i) duloxetine (832 patients), (ii) placebo (667 patients), or (iii) both duloxetine and placebo in a crossover analysis (41 patients) (Table 1) [20-24]. In the study by Skljarevski et al. (2009), patients were randomly assigned to receive duloxetine 20 mg daily, 60 mg daily, 120 mg daily, or placebo for 13 weeks. Patients assigned to receive duloxetine 60 mg or 120 mg daily were started with 30 mg daily and titrated up to 30 mg per week until the final dose was reached [20]. In the study by Skljarevski et al. (2010a), patients were randomly assigned to receive duloxetine or placebo for 13 weeks. In the duloxetine group the patients were given 30 mg daily for one week and 60 mg daily for 12 more weeks; however, for non-responders (a <30% reduction in BPI average pain response), the dose was increased to 120 mg daily for the last six weeks of the trial [21]. In the study by Skljarevski et al. (2010b), patients were randomly assigned to receive either duloxetine or placebo for 13 weeks. The duloxetine group received 60 mg daily for 12 weeks then 30 mg daily for a one-week taper [22]. In the study by Konno et al., patients were randomly assigned to receive duloxetine or placebo for 14 weeks. The duloxetine group received 20 mg daily for one week, 40 mg daily the following week, and 60 mg daily for the remaining 12 weeks [23]. In the study by Shuckro et al., patients were randomly assigned to receive either duloxetine or placebo for four weeks and were followed by a two-week washout period and a subsequent four-week crossover phase. In the duloxetine phase, the patients were titrated from 30 mg daily to 120 mg daily during the first two weeks and were maintained for the remaining two weeks [24]. All studies excluded patients with concomitant MDD. Three studies permitted the concomitant use of NSAIDs during the duration of the trial if the dosages were maintained during the trial [20-21,24].

The trial was completed by 625 patients in the duloxetine groups (75.1%) and 536 patients in the placebo groups (80.4%; p = 0.160) (Table 2). Twenty-one (51.2%) patients in the crossover study completed the trial. One-hundred-fifteen (115) patients in the duloxetine groups (13.8%) and 36 patients in the placebo groups (5.4%) discontinued the study due to adverse effects (p<0.001).

**Table 2:** Patient disposition NR = not recorded *p<0.05 versus placebo

Study		Skljarevski et al. 2009				Skljarevski et al. 2010a		Skljarevski et al. 2010b		Konno et al. 2016		Shuckro et al. 2017			
Groups		Duloxetine 20 mg	Duloxetine 60 mg	Duloxetine 120 mg	Placebo	Duloxetine 60-120 mg	Placebo	Duloxetine 60 mg	Placebo	Duloxetine 60 mg	Placebo	Duloxetine 120 mg Phase I	Placebo Phase I	Duloxetine 120 mg Phase II	Placebo Phase II
No. of Subjects		59	116	112	117	115	121	198	203	232	226	16	18	15	11
Trial completers, n (%)		43 (72.9)	80 (69.0)	62 (55.4)*	82 (70.1)	84 (73.0)	98 (81.0)	147 (74.2)	156 (76.8)	209 (90.1)	200 (88.5)	11 (68.8)	16 (88.9)	14 (93.3)	7 (63.6)
Discontinuations for any reason, n (%)		16 (27.1)	36 (31.0)	50 (44.6)*	35 (29.9)	31 (27.0)	23 (19.0)	51 (25.8)	47 (23.2)	23 (9.9)	26 (11.5)	5 (31.3)	2 (11.1)	1 (6.7)	4 (36.4)
	Adverse event	9 (15.3)	17 (14.7)	27 (24.1)*	10 (8.5)	16 (13.9)*	7 (5.8)	30 (15.2)*	11 (5.4)	16 (6.9)	8 (3.5)	5 (31.3)	2 (11.1)	1 (6.7)	4 (36.4)
	Lack of efficacy	2 (3.4)	4 (3.4)	5 (4.5)	6 (5.1)	0 (0.0)	1 (0.8)	1 (0.5)*	9 (4.4)	1 (0.4)	3 (1.3)	0 (0.0)	0 (0.0)	0 (0.0)	0 (0.0)
	Subject decision	3 (5.1)	6 (5.2)	6 (5.4)	14 (12.0)	NR	NR	6 (3.0)	5 (2.5)	4 (1.7)	10 (4.4)	0 (0.0)	0 (0.0)	0 (0.0)	0 (0.0)
	Lost to follow-up	1 (1.7)	6 (5.2)	5 (4.5)	2 (1.7)	NR	NR	NR	NR	0 (0.0)	0 (0.0)	0 (0.0)	0 (0.0)	0 (0.0)	0 (0.0)
	Protocol violation	0 (0.0)	3 (2.6)	4 (3.6)	3 (2.6)	NR	NR	NR	NR	NR	NR	0 (0.0)	0 (0.0)	0 (0.0)	0 (0.0)
	Physician decision	1 (1.7)	0 (0.0)	3 (2.7)	0 (0.0)	NR	NR	NR	NR	NR	NR	0 (0.0)	0 (0.0)	0 (0.0)	0 (0.0)
	Exclusion criteria met	NR	NR	NR	NR	NR	NR	NR	NR	1 (0.4)	1 (0.4)	0 (0.0)	0 (0.0)	0 (0.0)	0 (0.0)
	Other	0 (0.0)	0 (0.0)	0 (0.0)	0 (0.0)	15 (13.0)	15 (12.4)	14 (7.1)	22 (10.8)	1 (0.4)	4 (1.8)	0 (0.0)	0 (0.0)	0 (0.0)	0 (0.0)

Five studies containing 785 duloxetine patients (94.4%), 628 placebo patients (94.2%), and 25 crossover patients (61.0%) reported efficacy outcomes during the trial (Table 3). Four studies reported outcome scores including Brief Pain Inventory-Severity (BPI-S) scale, Brief Pain Inventory-Improvement (BPI-I) scale, Clinical Global Impressions of Severity (CGI-S), short-form 36 (SF-36), and Roland-Morris Disability Questionnaire (RMDQ-24) [20-23]. One study reported outcome scores including the visual analog scale (VAS) and painDETECT scores [24]. All five studies reported statistically significant improvements in more than one back-pain-specific clinical outcome score with duloxetine versus placebo. With respect to specific dosages, four studies found that duloxetine 60 mg daily leads to one or more statistically significant improvements versus placebo in BPI-S [20-23]. One study found that duloxetine 120 mg daily leads to significant improvement in visual analog scale and painDETECT scores versus placebo [24]. One study found no difference in back pain improvement with duloxetine 20 mg daily versus placebo (Table 3) [20].

**Table 3:** Efficacy outcomes ITT = intension-to-treat; PP = per protocol; BPI-S = Brief Pain Inventory-Severity scale; avg = average; BL = baseline; SD = standard deviation; NR = not recorded; f/u = follow-up; SE = standard error; BPI-I = Brief Pain Inventory-Interference scale; PGI-S = Patient’s Global Impressions of Severity; PGI-I = Patient’s Global Impressions of Improvement; CGI-S = Clinical Global Impressions of Severity; VAS = visual analog scale; NS = not significant; SF-36 = Short Form-36; MCS = mental composite score; PCS = physical composite score; RMDQ-24 = Roland-Morris Disability Questionnaire; EQ-5D = 5-dimension EuroQoL questionnaire; WPAI = Work Productivity and Activity Impairment Instrument *p<0.05 versus placebo

Study		Skljarevski et al. 2009				Skljarevski et al. 2010a		Skljarevski et al. 2010b		Konno et al. 2016		Shuckro et al. 2017			
Groups		Duloxetine 20 mg	Duloxetine 60 mg	Duloxetine 120 mg	Placebo	Duloxetine 60-120 mg	Placebo	Duloxetine	Placebo	Duloxetine 60 mg	Placebo	Duloxetine 120 mg ITT	Placebo ITT	Duloxetine 120 mg PP	Placebo PP
								60 mg							
No. of Subjects		59	116	112	117	115	121	198	203	232	226	11	14	7	14
Duration (weeks)		13	13	13	13	13	13	13	13	14	14	4	4	4	4
BPI-S avg pain BL, mean ± SD (n)		6.3 ± 1.6 (56)	5.9 ± 1.7 (108)	6.0 ± 1.6 (108)	6.1 ± 1.7 (113)	5.9 ± 1.6 (109)	6.0 ± 1.7 (116)	5.8 ± 1.4 (195)	5.8 ± 1.4)	5.14 ± 1.11 (230)	5.09 ± 1.04 (226)	NR	NR	NR	NR
BPI-S change at final f/u, mean ± SE (n)															
	Average pain	-1.79 ± 0.30 (56)	-2.50 ± 0.22 (108)*	-2.45 ± 0.22 (108)	-1.87 ± 0.22 (113)	-2.08 ± 0.20 (109)	-1.30 ± 0.19 (116)	0	-1.65 ± 0.15 (199)	-2.43 ± 0.11 (209)*	-1.96 ± 0.11 (200)	NR	NR	NR	NR
	Worst pain	-1.78 ± 0.35 (56)	-2.77 ± 0.25 (108)	-2.78 ± 0.26 (108)	-2.09 ± 0.25 (113)	-2.66 ± 0.23 (109)*	-1.90 ± 0.23 (116)	-2.56 ± 0.17 (195)*	-1.88 ± 0.17 (199)	-2.63 ± 0.13 (209)	-2.33 ± 0.13 (200)	NR	NR	NR	NR
	Least pain	-1.30 ± 0.29 (56)	-2.06 ± 0.21 (108)	-2.16 ± 0.21 (108)*	-1.51 ± 0.20 (113)	-1.70 ± 0.20 (109)*	-0.86 ± 0.19 (116)	-1.51 ± 0.14 (195)*	-1.02 ± 0.14 (199)	-1.69 ± 0.10 (209)*	-1.19 ± 0.11 (200)	NR	NR	NR	NR
	Pain right now	-1.63 ± 0.33 (56)	-2.67 ± 0.24 (108)	-2.61 ± 0.24 (108)*	-1.74 ± 0.24 (113)	-2.35 ± 0.24 (109)*	-1.42 ± 0.23 (116)	-2.40 ± 0.15 (195)*	-1.54 ± 0.15 (199)	-2.42 ± 0.12 (209)*	-2.03 ± 0.12 (200)	NR	NR	NR	NR
BPI-I change at final f/u, mean ± SE (n)															
	General activity	-1.99 ± 0.33 (56)	-2.52 ± 0.24 (107)	-2.36 ± 0.25 (108)	-1.97 ± 0.24 (113)	-2.09 ± 0.26 (109)	-1.49 ± 0.25 (115)	-2.36 ± 0.16 (195)*	-1.61 ± 0.16 (199)	-2.46 ± 0.13 (209)	-2.16 ± 0.13 (200)	NR	NR	NR	NR
	Mood	-1.75 ± 0.30 (56)	-2.52 ± 0.22 (107)*	-1.96 ± 0.22 (108)	-1.70 ± 0.21 (113)	-1.87 ± 0.27 (109)*	-0.96 ± 0.26 (115)	-1.98 ± 0.15 (195)*	-1.26 ± 0.15 (199)	-2.15 ± 0.11 (209)*	-1.83 ± 0.11 (200)	NR	NR	NR	NR
	Walking ability	-1.79 ± 0.34 (56)	-2.33 ± 0.25 (107)*	-1.89 ± 0.25 (108)	-1.43 ± 0.24 (113)	-2.04 ± 0.25 (109)*	-1.16 ± 0.24 (115)	-1.86 ± 0.15 (195)*	-1.40 ± 0.15 (199)	-2.05 ± 0.11 (209)	-1.92 ± 0.11 (200)	NR	NR	NR	NR
	Normal work	-2.20 ± 0.36 (56)	-2.67 ± 0.26 (107)*	-2.38 ± 0.26 (108)	-1.95 ± 0.26 (113)	-2.25 ± 0.26 (109)*	-1.50 ± 0.25 (115)	-2.17 ± 0.15 (195)*	-1.66 ± 0.15 (199)	-2.17 ± 0.12 (209)	-2.17 ± 0.12 (200)	NR	NR	NR	NR
	Relations with other people	-1.33 ± 0.27 (56)	-1.86 ± 0.20 (107)*	-1.27 ± 0.20 (108)	-0.94 ± 0.19 (113)	-1.59 ± 0.22 (109)*	-0.78 ± 0.22 (115)	-1.48 ± 0.15 (195)*	0.91 ± 0.14 (199)	-1.02 ± 0.10 (209)	-0.98 ± 0.10 (200)	NR	NR	NR	NR
	Sleep	-1.59 ± 0.32 (56)	-2.48 ± 0.24 (107)*	-2.12 ± 0.24 (108)	-1.63 ± 0.23 (113)	-1.95 ± 0.26 (109)	-1.31 ± 0.25 (115)	-2.14 ± 0.17 (195)*	-1.45 ± 0.17 (199)	-1.41 ± 0.11 (209)	-1.40 ± 0.11 (200)	NR	NR	NR	NR
	Enjoyment of life	-1.84 ± 0.32 (56)	-2.49 ± 0.24 (107)*	-1.86 ± 0.24 (108)	-1.76 ± 0.23 (113)	-1.74 ± 0.25 (109)*	-0.94 ± 0.24 (115)	-2.18 ± 0.15 (195)*	-1.61 ± 0.15 (199)	-1.52 ± 0.11 (209)	-1.48 ± 0.11 (200)	NR	NR	NR	NR
	Avg of 7 questions	-1.84 ± 0.26 (56)	-2.40 ± 0.19 (107)*	-1.92 ± 0.19 (108)	-1.61 ± 0.16 (113)	-1.92 ± 0.21 (109)*	-1.18 ± 0.20 (115)	-2.01 ± 0.13 (195)*	-1.43 ± 0.13 (199)	-1.83 ± 0.10 (209)	-1.70 ± 0.10 (200)	NR	NR	NR	NR
PGI-S at BL, mean ± SD		2.8 ± 1.7 (59)	2.6 ± 1.8 (116)	2.3 ± 1.6 (108)	2.4 ± 1.6 (117)	NR	NR	NR	NR	NR	NR	NR	NR	NR	NR
PGI-I at final f/u, mean ± SE (n)		2.71 (54)	2.43 (102)	2.65 (101)	2.92 (108)	2.59 (109)*	3.16 (115)	2.88 ± 0.09 (194)*	3.19 ± 0.09 (199)	2.46 ± 0.07 (209)*	2.76 ± 0.07 (200)	NR	NR	NR	NR
CGI-S at BL, mean ± SD		4.1 ± 1.4 (59)	3.5 ± 1.5 (116)	3.6 ± 1.3 (112)	3.7 ± 1.3 (117)	3.2 ± 1.5 (115)	3.2 ± 1.5 (121)	3.5 ± 1.2 (198)	3.3 ± 1.3 (203)	4.23 ± 0.66 (230)	4.22 ± 0.71 (226)	NR	NR	NR	NR
CGI-S change at final f/u, mean ± SE (n)		-0.53 ± 0.14 (58)	-0.94 ± 0.11 (108)*	-1.06 ± 0.11 (107)*	-0.53 ± 0.10 (112)	-0.98 ± 0.10 (110)	-0.77 ± 0.10 (117)	-0.95 ± 0.07 (195)	-0.79 ± 0.07 (199)	-1.46 ± 0.06 (209)*	-1.17 ± 0.06 (200)	NR	NR	NR	NR
VAS at final f/u, mean ± SD		NR	NR	NR	NR	NR	NR	NR	NR	NR	NR	4.1 ± 2.9*	6.0 ± 2.7	3.7 ± 2.9*	5.7 ± 2.5
VAS change at final f/u, mean ± SD		NR	NR	NR	NR	NR	NR	NR	NR	NR	NR	-2.7 ± 2.5*	-0.5 ± 1.6	NR	NR
painDETECT at final f/u, mean ± SD		NR	NR	NR	NR	NR	NR	NR	NR	NR	NR	NR	NR	17.7 ± 5.7*	21.3 ± 3.6
Athens Insomnia Scale change at final f/u, mean ± SE (n)		-1.43 ± 0.53 (54)	-2.30 ± 0.39 (101)*	-0.93 ± 0.40 (99)	-1.23 ± 0.38 (107)	-2.07 ± 0.047 (104)	-1.49 ± 0.47 (106)	NR	NR	NR	NR	NR	NR	NR	NR
SF-36 change at final f/u, mean ± SE (n)															
	Bodily pain	1.51 ± 0.27 (54)	1.95 ± 0.20 (102)*	2.11 ± 0.20 (101)*	1.36 ± 0.19 (108)	1.58 (109)*	1.04 (115)	16.28 ± 1.45 (188)*	11.70 ± 1.44 (190)	12.56 ± 0.94 (230)	11.01 ± 0.95 (226)	NR	NR	NR	NR
	General health	0.70 ± 0.41 (54)	1.24 ± 0.30 (102)	0.81 ± 0.30 (101)	0.66 ± 0.29 (108)	1.90 (109)*	0.87 (115)	6.96 ± 1.20 (188)	4.38 ± 1.20 (190)	6.72 ± 0.85 (230)*	3.78 ± 0.86 (226)	NR	NR	NR	NR
	Mental health	0.21 ± 0.49 (54)	0.98 ± 0.36 (102)	0.46 ± 0.36 (101)	0.38 ± 0.35 (108)	NS	NR	5.83 ± 1.07 (165)*	0.95 ± 1.07 (166)	5.63 ± 0.81 (230)*	2.42 ± 0.82 (226)	NR	NR	NR	NR
	Physical functioning	1.80 ± 0.52 (54)	2.55 ± 0.38 (102)	3.11 ± 0.38 (101)	2.23 ± 0.37 (108)	NS	NR	11.67 ± 1.40 (186)	8.18 ± 1.41 (189)	8.47 ± 0.79 (230)	7.20 ± 0.80 (226)	NR	NR	NR	NR
	Role-emotional	0.10 ± 0.12 (54)	0.19 ± 0.09 (102)	0.14 ± 0.09 (101)	0.08 ± 0.09 (108)	NS	NR	6.81 ± 1.77 (172)	4.39 ± 1.76 (179)	5.78 ± 1.13 (230)	6.18 ± 1.14 (226)	NR	NR	NR	NR
	Role-physical	0.81 ± 0.21 (54)	0.80 ± 0.15 (102)	0.85 ± 0.15 (101)	0.80 ± 0.15 (108)	NS	NR	10.03 ± 1.93 (172)	8.12 ± 1.92 (179)	10.58 ± 1.15 (230)	10.00 ± 1.16 (226)	NR	NR	NR	NR
	Social functioning	0.75 ± 0.21 (54)	0.46 ± 0.16 (102)	0.38 ± 0.16 (101)	0.50 ± 0.15 (108)	NS	NR	11.50 ± 1.40 (188)*	7.51 ± 1.40 (190)	6.40 ± 1.00 (230)	4.77 ± 1.01 (226)	NR	NR	NR	NR
	Vitality	0.69 ± 0.50 (54)	1.43 ± 0.36 (102)	0.44 ± 0.37 (101)	0.91 ± 0.35 (108)	1.46 (109)*	0.43 (115)	8.73 ± 1.36 (163)*	4.63 ± 1.35 (165)	5.56 ± 0.97 (230)	4.41 ± 0.97 (226)	NR	NR	NR	NR
SF-36 MCS at final f/u, mean ± SD (n)		NR	NR	NR	NR	NR	NR	NR	NR	NR	NR	NR	NR	50.0 ± 11.6*	46.5 ± 12.5
SF-36 PCS at final f/u, mean ± SD (n)		NR	NR	NR	NR	NR	NR	NR	NR	NR	NR	NR	NR	36.0 ± 10.9*	31.3 ± 9.3
RMDQ-24 change at f/u, mean ± SE (n)		-2.28 (54)	-2.74 (102)*	-2.88 (101)*	-1.33 (108)	-3.60 (109)*	-1.93 (115)	-2.69 ± 0.31 (178)	-2.22 ± 0.32 (179)	-3.86 ± 0.22 (230)*	-3.23 ± 0.22 (226)	NR	NR	NR	NR
EQ-5D change at final f/u, mean ± SE (n)		0.07 ± 0.03 (54)	0.11 ± 0.02 (102)	0.13 ± 0.02 (100)	0.08 ± 0.02 (104)	NR	NR	0.15 ± 0.02 (190)*	0.07 ± 0.02 (192)	0.09 ± 0.01 (230)	0.08 ± 0.01 (226)	NR	NR	NR	NR
WPAI change at final f/u, mean ± SE (n)															
	Work time missed	NR	NR	NR	NR	NS	NR	0.0 ± 0.0 (79)	-0.01 ± 0.02 (93)	-0.01 ± 0.01 (140)*	0.02 ± 0.01 (143)	NR	NR	NR	NR
	Impairment at work	NR	NR	NR	NR	NS	NR	-0.19 ± 0.03 (79)	-0.16 ± 0.02 (90)	-0.13 ± 0.02 (140)	-0.09 ± 0.02 (143)	NR	NR	NR	NR
	Work productivity loss	NR	NR	NR	NR	NS	NR	-0.18 ± 0.03 (77)	-0.16 ± 0.03 (89)	-0.13 ± 0.02 (140)	-0.09 ± 0.02 (143)	NR	NR	NR	NR
	Work activity impairment	NR	NR	NR	NR	NS	NR	-0.20 ± 0.02 (190)*	-0.15 ± 0.02 (196)	-0.14 ± 0.01 (230)	-0.12 ± 0.01 (226)	NR	NR	NR	NR
Global Impression of Improvement, n (%)															
	Improved	NR	NR	NR	NR	NR	NR	NR	NR	191 (83.0)*	163 (72.1)	NR	NR	NR	NR
	Unchanged	NR	NR	NR	NR	NR	NR	NR	NR	34 (14.8)*	59 (26.1)	NR	NR	NR	NR
	Worsened	NR	NR	NR	NR	NR	NR	NR	NR	5 (2.2)	4 (1.8)	NR	NR	NR	NR

All five studies reported incidences of serious and minor adverse events (AEs). All five studies found no significant difference in serious AEs between the duloxetine and placebo groups. Four studies found no significant difference in total AEs between the duloxetine and placebo groups (Table 4) [21-24]. One study found a higher rate of total AEs among the duloxetine 120 mg group (72.3%) vs the placebo group (59.0%); however, the same study did not find a significant difference in total AEs among duloxetine 20 mg and 60 mg groups versus placebo [20]. With respect to specific AEs, four studies reported a higher incidence of nausea, three studies reported a higher incidence of dry mouth, and two studies reported a higher incidence of constipation, fatigue, and somnolence with duloxetine versus placebo (Table 4). 

**Table 4:** Adverse events AE = adverse event; NR = not recorded *p<0.05 versus placebo

Study		Skljarevski et al. 2009				Skljarevski et al. 2010a		Skljarevski et al. 2010b		Konno et al. 2016		Shuckro et al. 2017	
Groups		Duloxetine 20 mg	Duloxetine 60 mg	Duloxetine 120 mg	Placebo	Duloxetine 60-120 mg	Placebo	Duloxetine 60 mg	Placebo	Duloxetine 60 mg	Placebo	Duloxetine Phase	Placebo Phase
No. of Patients		59	116	112	117	115	121	198	203	232	226	31	29
Total Pts with 1+ AE		38 (64.4)	78 (67.2)	81 (72.3)*	69 (59.0)	65 (56.5)	58 (47.9)	125 (63.1)	111 (55.1)	NR	NR	20 (64.5)	18 (62.1)
Serious AEs		1 (1.7)	1 (0.9)	3 (2.7)	3 (2.6)	4 (3.5)	1 (0.8)	5 (2.5)	0 (0.0)	4 (1.7)	4 (1.8)	0 (0.0)	0 (0.0)
	Non-cardiac chest pain	1 (1.7)	0 (0.0)	0 (0.0)	1 (0.9)	0 (0.0)	0 (0.0)	0 (0.0)	0 (0.0)	0 (0.0)	0 (0.0)	0 (0.0)	0 (0.0)
	Vertigo	0 (0.0)	0 (0.0)	0 (0.0)	1 (0.9)	0 (0.0)	0 (0.0)	1 (0.5)	0 (0.0)	0 (0.0)	0 (0.0)	0 (0.0)	0 (0.0)
	Peritonsillar abscess	0 (0.0)	0 (0.0)	0 (0.0)	1 (0.9)	0 (0.0)	0 (0.0)	0 (0.0)	0 (0.0)	0 (0.0)	0 (0.0)	0 (0.0)	0 (0.0)
	Dyspnea	0 (0.0)	1 (0.9)	0 (0.0)	0 (0.0)	0 (0.0)	0 (0.0)	0 (0.0)	0 (0.0)	0 (0.0)	0 (0.0)	0 (0.0)	0 (0.0)
	Hypertensive encephalopathy	0 (0.0)	0 (0.0)	0 (0.0)	0 (0.0)	1 (0.9)	0 (0.0)	0 (0.0)	0 (0.0)	0 (0.0)	0 (0.0)	0 (0.0)	0 (0.0)
	Perioral numbness	0 (0.0)	0 (0.0)	1 (0.9)	0 (0.0)	0 (0.0)	0 (0.0)	0 (0.0)	0 (0.0)	0 (0.0)	0 (0.0)	0 (0.0)	0 (0.0)
	Transient ischemic attack	0 (0.0)	0 (0.0)	1 (0.9)	0 (0.0)	1 (0.9)	0 (0.0)	0 (0.0)	0 (0.0)	0 (0.0)	0 (0.0)	0 (0.0)	0 (0.0)
	Myocardial infarction	0 (0.0)	0 (0.0)	1 (0.9)	0 (0.0)	0 (0.0)	1 (0.8)	1 (0.5)	0 (0.0)	0 (0.0)	0 (0.0)	0 (0.0)	0 (0.0)
	Osteoarthritis	0 (0.0)	0 (0.0)	0 (0.0)	0 (0.0)	1 (0.9)	0 (0.0)	0 (0.0)	0 (0.0)	0 (0.0)	1 (0.4)	0 (0.0)	0 (0.0)
	Wrist fracture	0 (0.0)	0 (0.0)	0 (0.0)	0 (0.0)	1 (0.9)	0 (0.0)	0 (0.0)	0 (0.0)	0 (0.0)	0 (0.0)	0 (0.0)	0 (0.0)
	Toxic myopathy	0 (0.0)	0 (0.0)	0 (0.0)	0 (0.0)	0 (0.0)	0 (0.0)	1 (0.5)	0 (0.0)	0 (0.0)	0 (0.0)	0 (0.0)	0 (0.0)
	Asthma	0 (0.0)	0 (0.0)	0 (0.0)	0 (0.0)	0 (0.0)	0 (0.0)	1 (0.5)	0 (0.0)	0 (0.0)	0 (0.0)	0 (0.0)	0 (0.0)
	Alcohol poisoning	0 (0.0)	0 (0.0)	0 (0.0)	0 (0.0)	0 (0.0)	0 (0.0)	1 (0.5)	0 (0.0)	0 (0.0)	0 (0.0)	0 (0.0)	0 (0.0)
	Pneumonia	0 (0.0)	0 (0.0)	0 (0.0)	0 (0.0)	0 (0.0)	0 (0.0)	0 (0.0)	0 (0.0)	0 (0.0)	2 (0.9)	0 (0.0)	0 (0.0)
	Cerebral hemorrhage	0 (0.0)	0 (0.0)	0 (0.0)	0 (0.0)	0 (0.0)	0 (0.0)	0 (0.0)	0 (0.0)	1 (0.4)	0 (0.0)	0 (0.0)	0 (0.0)
	Gastric polyps	0 (0.0)	0 (0.0)	0 (0.0)	0 (0.0)	0 (0.0)	0 (0.0)	0 (0.0)	0 (0.0)	1 (0.4)	0 (0.0)	0 (0.0)	0 (0.0)
	Urethral calculus	0 (0.0)	0 (0.0)	0 (0.0)	0 (0.0)	0 (0.0)	0 (0.0)	0 (0.0)	0 (0.0)	1 (0.4)	0 (0.0)	0 (0.0)	0 (0.0)
	Intervertebral disc protrusion	0 (0.0)	0 (0.0)	0 (0.0)	0 (0.0)	0 (0.0)	0 (0.0)	0 (0.0)	0 (0.0)	1 (0.4)	0 (0.0)	0 (0.0)	0 (0.0)
	Hemothorax	0 (0.0)	0 (0.0)	0 (0.0)	0 (0.0)	0 (0.0)	0 (0.0)	0 (0.0)	0 (0.0)	0 (0.0)	1 (0.4)	0 (0.0)	0 (0.0)
Minor AEs that occurred at incidence > 5%													
	Nausea	11 (18.6)*	24 (20.7)*	13 (11.6)*	4 (3.4)	13 (11.3)*	3 (2.4)	35 (17.7)*	5 (2.5)	21 (9.0)*	6 (2.7)	6 (19.4)	1 (3.4)
	Insomnia	5 (8.5)	10 (8.6)*	21 (18.8)*	3 (2.6)	NR	NR	NR	NR	NR	NR	2 (6.5)	4 (13.8)
	Dry mouth	3 (5.1)	12 (10.3)*	12 (10.7)*	1 (0.9)	10 (8.7)	4 (3.3)	13 (6.6)	3 (1.5)	14 (6.0)*	0 (0.0)	11 (35.5)*	1 (3.4)
	Constipation	2 (3.4)	10 (8.6)*	14 (12.5)*	1 (0.9)	6 (5.2)	1 (0.8)	12 (6.1)	6 (3.0)	25 (10.7)*	5 (2.2)	6 (19.4)	2 (6.9)
	Headache	2 (3.4)	11 (9.5)	10 (8.9)	4 (3.4)	4 (3.5)*	13 (10.7)	26 (13.1)	24 (11.8)	NR	NR	NR	NR
	Diarrhea	2 (3.4)	10 (8.6)	8 (7.1)	4 (3.4)	7 (6.1)	6 (5.0)	NR	NR	NR	NR	2 (6.5)	4 (13.8)
	Dizziness	3 (5.1)	9 (7.8)	9 (8.0)	3 (2.6)	6 (5.2)	2 (1.7)	10 (5.1)	2 (1.0)	15 (6.4)*	2 (0.9)	5 (16.1)	3 (10.3)
	Somnolence	3 (5.1)*	5 (4.3)*	14 (12.5)*	0 (0.0)	NR	NR	NR	NR	45 (19.2)*	16 (7.1)	NR	NR
	Fatigue	0 (0.0)	7 (6.0)*	10 (8.9)*	0 (0.0)	8 (7.0)*	1 (0.8)	NR	NR	NR	NR	8 (25.8)	2 (6.9)
	Hyperhidrosis	NR	NR	NR	NR	7 (6.1)*	0 (0.0)	NR	NR	NR	NR	11 (35.5)	8 (27.6)
	Nasopharyngitis	NR	NR	NR	NR	NR	NR	NR	NR	26 (11.1)	39 (17.4)	NR	NR
	Contusion	NR	NR	NR	NR	NR	NR	NR	NR	16 (6.8)	7 (3.1)	0 (0.0)	0 (0.0)
	Appetite loss	NR	NR	NR	NR	NR	NR	NR	NR	NR	NR	6 (19.4)*	0 (0.0)
	Increased pain	NR	NR	NR	NR	NR	NR	NR	NR	NR	NR	0 (0.0)*	4 (13.8)

All five studies reported incidences of changes in vital signs and laboratory values, including complete blood count (CBC) and comprehensive metabolic panel (CMP) (Table 5). Two studies reported a higher heart rate and more weight loss and one study reported a higher diastolic blood pressure with duloxetine versus placebo [20-21]. However, neither of the reported changes was determined to be clinically significant. There were no significant differences in the remaining vital signs (systolic blood pressure, temperature) or blood chemistry values (CBC, CMP) between the two groups among all five studies.

**Table 5:** Vitals and laboratory values HR = heart rate; SE = standard error; NS = not significant; NR = not recorded; SBP = systolic blood pressure; DBP = diastolic blood pressure; CMP = comprehensive metabolic panel; CBC = complete blood count *p<0.05 versus placebo

Study		Skljarevski et al. 2009				Skljarevski et al. 2010a		Skljarevski et al. 2010b		Konno et al. 2016		Shuckro et al. 2017	
Groups		Duloxetine 20 mg	Duloxetine 60 mg	Duloxetine 120 mg	Placebo	Duloxetine 60-120 mg	Placebo	Duloxetine 60 mg	Placebo	Duloxetine 60 mg	Placebo	Duloxetine Phase	Placebo Phase
No. of Patients		59	116	112	117	115	121	198	203	232	226	31	29
Vitals													
	Change in HR, mean (SE)	NS	2.79 (0.89)*	NS	0.29 (0.87)	2.38 (1.09)*	-0.76 (1.08)	NS	NR	NS	NR	NS	NR
	Change in SBP, mean (SE)	NS	NS	NS	NR	NS	NR	NS	NR	NS	NR	NS	NR
	Change in DBP, mean (SE)	NS	NS	2.94 (0.85)*	-0.68 (0.82)	NS	NR	NS	NR	NS	NR	NS	NR
	Change in temperature, (C^o^) mean (SE)	NS	NS	NS	NR	NS	NR	NS	NR	NS	NR	NS	NR
	Change in weight (kg), mean (SE)	NS	NS	-0.72 (0.22)*	0.10 (0.22)	-0.49 (0.21)*	0.24 (0.21)	NS	NR	NS	NR	NS	NR
Blood chemistry (CMP, CBC)		No significant difference amongst all values				No significant difference amongst all values		No significant difference amongst all values		No significant difference amongst all values		No significant difference amongst all values	

Discussion

To our knowledge, this is the first comprehensive systematic review of randomized, placebo-controlled trials that investigates the safety and efficacy of duloxetine for CLBP.

The authors hypothesized that duloxetine is a safe and effective pharmacological intervention for the treatment of CLBP. The extracted evidence supported the first hypothesis, as all five included studies found no clinically significant difference in vital signs, blood chemistry values, or serious AEs. Although one study found a higher rate of total minor AEs, this was specifically in the duloxetine 120 mg group and not within the 20 or 60 mg groups. The evidence also supported the second hypothesis, as all five studies reported statistically significant improvements in more than one back-pain-specific clinical outcome score with duloxetine versus placebo.

Although all analyzed studies demonstrated a more substantial improvement in one or more back-pain-specific outcome scores with the use of duloxetine versus placebo, these studies were limited by failing to compare individual outcome scores with the minimal clinically important difference (MCID) scores. An analysis by Mease et al. that pooled the data of four randomized, double-blind, placebo-controlled trials of duloxetine for the treatment of fibromyalgia, with a total of 1411 subjects, determined that the MCID for the BPI-S average pain and BPI-S severity subscores are 2.1 and 2.2, respectively [34]. The mean BPI-S average pain subscore improvement reported by the duloxetine 60 mg groups by Skljarevski et al. 2009, Skljarevski et al. 2010b, and Konno et al. 2016 within our study were 2.50 ± 0.22, 2.25 ± 0.15, and 2.43 ± 0.11, respectively [20,22-23]. Although Skljarevski et al. 2010a did not report a statistically significant BPI-S average pain subscore improvement (2.08 ± 0.20), they reported a statistically significant mean BPI-S worst pain and pain right now severity subscore improvement of 2.66 ± 0.23 and 2.35 ± 0.24, respectively. Thus, though individual scores were not reported, each reported mean exceeded the MCID reported by Mease et al., which signifies that a majority of the patients within the duloxetine 60 mg groups experienced both a statistically significant and clinically relevant improvement in either the BPI-S average pain or severity subscores [34].

A study by Ostelo et al. found that the MCID for VAS CLBP is 2.5 [35]. Thus, the crossover study by Shuckro et al., reporting a mean VAS improvement of 2.7± 2.5, may signify that a majority of the patients receiving duloxetine 120 mg had both a statistically significant and a clinically relevant improvement in VAS compared to placebo. Other reported MCIDs in the literature consist of 1.9 for the Athens Insomnia Scale (AIS), 4.0 for the SF-36 physical composite score (PCS), 3.5 for RMDQ, and 0.08 for the five-dimension EuroQoL questionnaire (EQ-5D) [35-38]. In comparison, one study within this analysis reported a mean AIS improvement of 2.3 within the duloxetine 60 mg group, one study reported a mean SF-36 PCS improvement of 4.7, two studies reported a mean RMDQ-24 improvement of 3.6 and 3.86, and one study reported a mean EQ-5D improvement of 0.15. These results demonstrate that a majority of the patients receiving duloxetine in all five included studies experienced both a statistically significant and clinically relevant improvement in more than one back-pain-specific outcome score. Another factor that is of note is the potential role weight loss may have had on CLBP changes and whether it was an adverse outcome of SNRI or from lifestyle changes, as two studies in the analysis reported significant weight loss in the duloxetine 60 mg and 60-120 mg groups. 

NSAIDs are currently indicated as the first-line pharmacological treatment for non-specific CLBP [10]. However, a recent Cochrane review by Enthoven et al. demonstrated that merely six of 13 randomized controlled trials included in the review showed that NSAIDs are more effective than placebo in regard to pain improvement [39]. The same study reported an overall improvement of back pain VAS of 0.697, which is significantly lower than the VAS improvement reported within our systematic review (2.7 ± 2.5 by Schukro et al.) [24]. A systematic review by Castellsague et al. also reported a higher risk of gastrointestinal complications associated with NSAIDs as compared to placebo, particularly when taken chronically or with high doses [40]. Furthermore, a meta-analysis by Aweid et al. reported that NSAIDs, particularly ibuprofen and celecoxib, are associated with high renal and cardiovascular complications, respectively [41]. Our systematic review found no differences in gastrointestinal, renal, or cardiovascular complications between the intervention and placebo groups. On the contrary, two studies within our analysis reported a higher drop-out rate among the duloxetine group due to minor AEs [20,22]. However, these patients were assigned to the 120 mg groups and patients taking 60 mg or less resulted in similar drop-out and rates of AEs compared with placebo. These results demonstrate that 60 mg taken once daily has the highest efficacy for reducing pain and disability while minimizing minor adverse effects.

Opioids, including tramadol, are often used as a second-line pharmacological treatment for non-specific CLBP [14-15]. A meta-analysis by Petzke et al. that investigated 21 studies with 7650 participants reported a clinically relevant reduction of pain without significant adverse events with the short-term use (up to 15 weeks) of opioids for CLBP [42]. However, a systematic review by Vowles et al. that investigated opioid use in chronic pain reported rates of addiction and misuse of up to 17% and 38%, respectively [43]. In comparison, there were no reports of misuse or addiction associated with the use of duloxetine within our systematic review.

There are limitations to this systematic review. First, the heterogeneity of the included studies with variable inclusion criteria, outcome measures, and follow-up time precluded a meta-analysis and limited direct comparisons of results. Although high-quality comparative studies were included in this review, only one study was level I evidence. Furthermore, none of the included studies reported minimal clinically important difference (MCID) scores to compare individual differences in outcomes scores; thus, the clinical relevance of the statistically significant difference in the reported outcomes scores remains largely unknown. Also, studies may have included patients with CLBP with an underlying condition like fibromyalgia, which SRNIs are indicated for. Lastly, it is possible that our stringent search protocol and limiters may have excluded other relevant studies on this topic, including those published in the non-English language.

## Conclusions

Duloxetine is a safe and effective first-line option for the treatment of CLBP. Current studies demonstrate that 60 mg taken once daily has the highest efficacy for reducing pain and disability while minimizing minor adverse effects. Further randomized controlled trials with long-term follow-up are necessary to determine its long-term effects.
